# Self-reported screening practices of family physicians participating in the colorectal cancer screening program of the canton of Vaud: a cross-sectional study

**DOI:** 10.1186/s12875-020-01176-z

**Published:** 2020-06-10

**Authors:** Camille Poroes, Jacques Cornuz, Alexandre Gouveia, Cyril Ducros, Kevin Selby

**Affiliations:** 1 Les Ligues pour la santé du canton de Fribourg, Fribourg, Switzerland; 2 Center for Primary Care and Public Health (Unisanté), University of Lausanne, Rue de Bugnon 44, 1011 Lausanne, Switzerland

**Keywords:** Family physician, Colorectal cancer, Screening program, Screening methods, Colonoscopy, Fit

## Abstract

**Background:**

The organized colorectal cancer (CRC) screening program in the canton of Vaud, Switzerland offers citizens the choice of the faecal immunochemical test (FIT) or colonoscopy via a visit with a family physician (FP). Given the central role of FPs in the program, this study aimed to compare their self-reported preventive practices with the objectives of the program, namely to inform patients about CRC screening and present the choice of colonoscopy and FIT, and to identify factors associated with presenting a choice of tests.

**Methods:**

Mixed-methods study using an online survey and semi-structured interviews. Participants were FPs from the canton of Vaud who had included ≥1 patient in the screening program. We used multivariate logistic regression to compare FPs offering only colonoscopy to those who offered a choice of tests or FIT.

**Results:**

The participation rate was 40% (177 respondents / 443 eligible). Most FPs (68%) reported informing more than 75% of eligible patients about the program. Lack of time (*n* = 86, 33%) was the principal reason cited for not informing patients. Regarding the screening methods, 20% (*n* = 36) of FPs prescribed only colonoscopy, 13% (*n* = 23) only FIT and 65% (*n* = 115) both screening methods. Predictors of offering only colonoscopy rather than a choice of screening tests included: first, FP reporting that they chose/would choose colonoscopy for themselves (OR 8.54 [95% CI 1.83–39.79, *P* < 0.01]); second, being > 20 years in practice (OR 4.8 [95% CI 1.3–0.17.66, *P* = 0.02]); and third, seeing 300 or more patients per month (OR 3.05 [95% CI 1.23–7.57, *P* = 0.02]). When asked what could improve the program, 17% (*n* = 31) wrote that patients should be informed in advance about the program by postal mail and a large-scale communication campaign.

**Conclusion:**

The majority of FPs reported CRC screening practices consistent with the objectives of the program. However, to ensure that patients are well informed and to save time, all patients need to be systematically informed about the program. Further, FPs should be encouraged to offer a choice of tests.

## Background

The burden of colorectal cancer (CRC) in Switzerland is substantial, with 4100 new cases per year [1]. Colorectal cancer meets the criteria defined by the WHO for the establishment of an organized screening program [2]. Population-based studies show the decrease in mortality is about 25% with the Faecal Occult Blood Test (meta-analysis of randomized trials), with even greater effects expected with the Faecal Immunochemical Test (FIT), and between 37 and 77% decrease with colonoscopy (case-control studies) [3–7].

A screening program using semi-quantitative faecal immunochemical testing (FIT) and screening colonoscopy was implemented in canton of Vaud, Switzerland in 2015 [8]. The aim of the program is to offer CRC screening to all men and women aged 50 to 69 years residing in the canton of Vaud and to facilitate their choice of screening test [9]. Invitation letters with a decision aid describing CRC screening are sent by post to eligible men and women aged 50 to 69 years. Inclusion in the program can be achieved via FPs either after receiving this mailed invitation or if CRC screening is brought up spontaneously by the patient or the FP during a medical consultation [9] [10]. In order for FPs to be able to share the patient’s personal data with the Fondation vaudoise pour le dépistage du cancer (FVDC) and thus include the patient in the program, two options are offered: on the program’s portal site or a paper inclusion form. The medical consultation to include the patient in the program, as well as the screening test itself, are covered by basic health insurance without financial deductible. There is still a 10% share of the cost of the people participating in the screening program, which means for FIT, approximately 4 CHF, and 80 CHF for a colonoscopy [10].

It is widely recognized that, in addition to having an essential role in primary prevention, FPs are key partners in screening programs, especially CRC screening. The active participation of FPs increases the successful implementation of a screening intervention [11]. They are the intermediaries between the screening centre and the target population and are responsible for monitoring the inclusion and exclusion criteria of people in the program [12, 13]. Furthermore, population participation rates are higher if FPs recommend screening to their patients. Proposing both screening methods or FIT only can improve participation as opposed to recommending only colonoscopy [14, 15].

The role of FPs in the CRC screening program deployed in the canton of Vaud is critical to its success. Given that no study had been conducted on their actual involvement in this public health intervention 3 years after its launch, we conducted an evaluation of their role and level of satisfaction. The aim of the present study was to compare the preventive practices of FPs involved in the CRC screening program with the objectives of the program, specifically “informing patients of the program” and “presenting the choice of colonoscopy and FIT”, and to explain the convergence or non-convergence between program objectives and current practice.

## Method

We performed a mixed-methods assessment of FPs’ involvement in the CRC screening program in the canton of Vaud using quantitative data from an online survey and qualitative data from semi-structured interviews. Ethics approval is not required in the canton of Vaud for anonymously collected data of health professionals [16]. However, participants had the option of participating in the online survey or not. For face-to-face interviews, they gave their consent during the survey by giving their email address to contact them.

Participants were FPs from the canton of Vaud who had included at least one patient in the CRC screening program since 2015. In 2016 there were 512 FPs registered to practice in the canton of Vaud [17]. At the time of the current study, 458 (89%) had ratified an agreement with the FVDC [10], of whom 443 (97%) had an email address available.

The survey consisted of 28 questions and was created on surveygizmo.com (supplementary files). The internet link was sent on 08 June 2018 to each FP’s professional e-mail address through the FVDC with a 20-day deadline to reply. A reminder e-mail was sent on 19 June 2018. Our primary outcomes were FP’s responses to the question “What proportion of your patients aged 50 to 69 do you inform about the screening program during their first 3 consultations?” and “What screening method do you offer?” We also collected information about FP’s views of the program, patients included to date, personal prevention practices, and FP’s practice characteristics. The final question in the survey was whether the FP agreed to be contacted again for an interview. If they agreed, they could give their e-mail address. The survey was closed on 29 June 2018.

To evaluate whether the objectives proposed by the program had been achieved, we defined a priori the minimum target to be reached to fulfil the program objectives: more than 50% of FPs inform 75% or more of their eligible patients; and more than 50% of FPs offer both screening methods. The 35 FPs who provided their e-mail address during the online survey were contacted for an interview. Ten face-to-face meetings were scheduled during July 2018. The remaining 25 FPs were not interviewed due to non-response on their part or unavailability during the time provided. Semi-directive interviews, lasting ~ 20 min were conducted using an interview grid (supplemental materials). We asked about: general preventive practices, response to a vignette of a 55- year-old male who was going to his doctor for back pain due to osteoarthritis, who smoked one pack a day and had no high risk criteria for CRC; information about the program; approach to CRC screening and screening methods used; and finally, their opinion about the program.

### Statistical analysis

Data were analysed anonymously and compared with the objectives of the program. The quantitative data from the survey were exported from the surveygizmo.com platform to Stata. Data are presented as numbers and percentages. First, descriptive analyses were used to present FPs’ characteristics. Univariate and multivariate logistic regressions were used to find associations between FPs’ characteristics and first, whether they informed > 75% of their patients, and second, whether they reported offering only colonoscopy vs. a choice of screening tests. We chose to dichotomize these variables in this sense because proposing both screening methods or FIT alone can improve participation, as opposed to recommending colonoscopy alone, which can reduce adherence to CRC screening [14, 15]. Odds ratios (OR) with 95% confidence intervals (95% CI) were reported as an estimate of the magnitude of association. Statistical significance was set at a *p*-value of less than 0.05.

### Qualitative analysis

A thematic analysis was used to interpret qualitative data. The qualitative data obtained through the semi-structured interviews were analysed to highlight the main and recurring themes. We created a content analysis grid for each interview in which, under each theme, the extracts or quotations from the interview were written. The overlapping excerpts or quotations have been grouped into different categories. All content analysis workbooks were gathered to count the number of similar points of view.

## Results

Of 443 FPs invited, 239 did not complete any part of the survey, 27 responses were incomplete and therefore not eligible, leaving a total of 177 complete responses (40% participation). Table 1 describes the socio-demographic characteristics of the 177 FPs who completed the survey and the results of the logistic regression.

**Table 1:** Physician characteristics, stratified by those offering colonoscopy only and those both FIT and colonoscopy or FIT alone (*n* = 178)

	Total (*n* = 178)	Number of physicians offering colonoscopy only (*n* = 36)	Number of physicians offering both methods or FIT alone (*n* = 142)	Multivariate odds ratio (95% conf. interval) *
**Practice location**				
Rural	26	4 (11.1%)	22 (15.5%)	
Urban	151	32 (88.9%)	119 (83.8%)	
Other	1		1 (0.7%)	
**Years in practice**				
Less than 20 years	111	12 (33.4%)	99 (69.7%)	OR 4.8 (95% CI 1.3–0.17.66, *P* = 0.02)
More than 20 years	67	24 (66.6%)	43 (30.3%)	
**FP Activity rate**				
Less than 50%	12	1 (2.8%)	11 (7.8%)	
More than 50%	166	35 (97.2%)	131 (92.2%)	
**Number of patients seen per month**				
Less than 300	103	14 (38.9%)	89 (62.7%)	OR 3.05 (95% CI 1.23–7.57, P = 0.02)
More than 300	75	22 (61.1%)	53 (37.3%)	
**Gender**				
Male	101	24 (66.6%)	77 (54.3%)	
Female	75	12 (33.4%)	63 (44.3%)	
Other	2		2 (1.4%)	
**Age**				
Less than 50 years old	81	9 (25%)	72 (50.7%)	OR 0.67 (95% CI 0.17–2.63)
More than 50 years old	97	27 (75%)	70 (49.3%)	
**Choice for own screening**				
FIT	49	2 (5.5%)	47 (33.1%)	OR 8.54 (95% CI 1.83–39.79, P < 0.01)
Colonoscopy	104	29 (80.6%)	75 (52.8%)	
Other	25	5 (13.9%)	20 (14.1%)	
**Participation in training sessions**				
No	90	24 (66.6%)	66 (46.5%)	OR 0.58 (95% CI 0.23–1.45)
Yes	88	12 (33.4%)	76 (53.5%)	
**Physical Activity**				
Less than 3 times per month	25	1 (2.8%)	24 (16.9%)	
More than 3 times per month	142	30 (83.4%)	112 (78.9%)	
Other	11	5 (13.8%)	6 (4.2%)	
**Alcohol consumption**				
Never	16	2 (5.6%)	14 (9.8%)	
Sometimes or regular	154	30 (83.4%)	124 (87.4%)	
Other	8	4 (11%)	4 (2.8%)	
**Tabaco**				
No-smoker	147	29 (80.6%)	118 (83.1%)	
Smoker	23	4 (11%)	19 (13.4%)	
Other	8	3 (8.4%)	5 (3.5%)	
**Food type**				
Salty, fatty and sweet diet	4	0 (0%)	4 (2.8%)	
Diversified diet	165	33 (91.6%)	132 (93%)	
Other	9	3 (8.4%)	6 (4.2%)	

*Only variables significant to *p* ≤ 0.05 in univariate analyses were included in the multivariate model

Two-thirds of FPs (*n* = 121, 68%) informed between 75 and 100% of their eligible patients of the existence of the program within the first 3 consultations. 26% (*n* = 45) of FPs had included between 1 and 5 patients in the program in the past 6 months and 70% (*n* = 124) of FPs had included more than 5 patients.

**Fig. 1:**
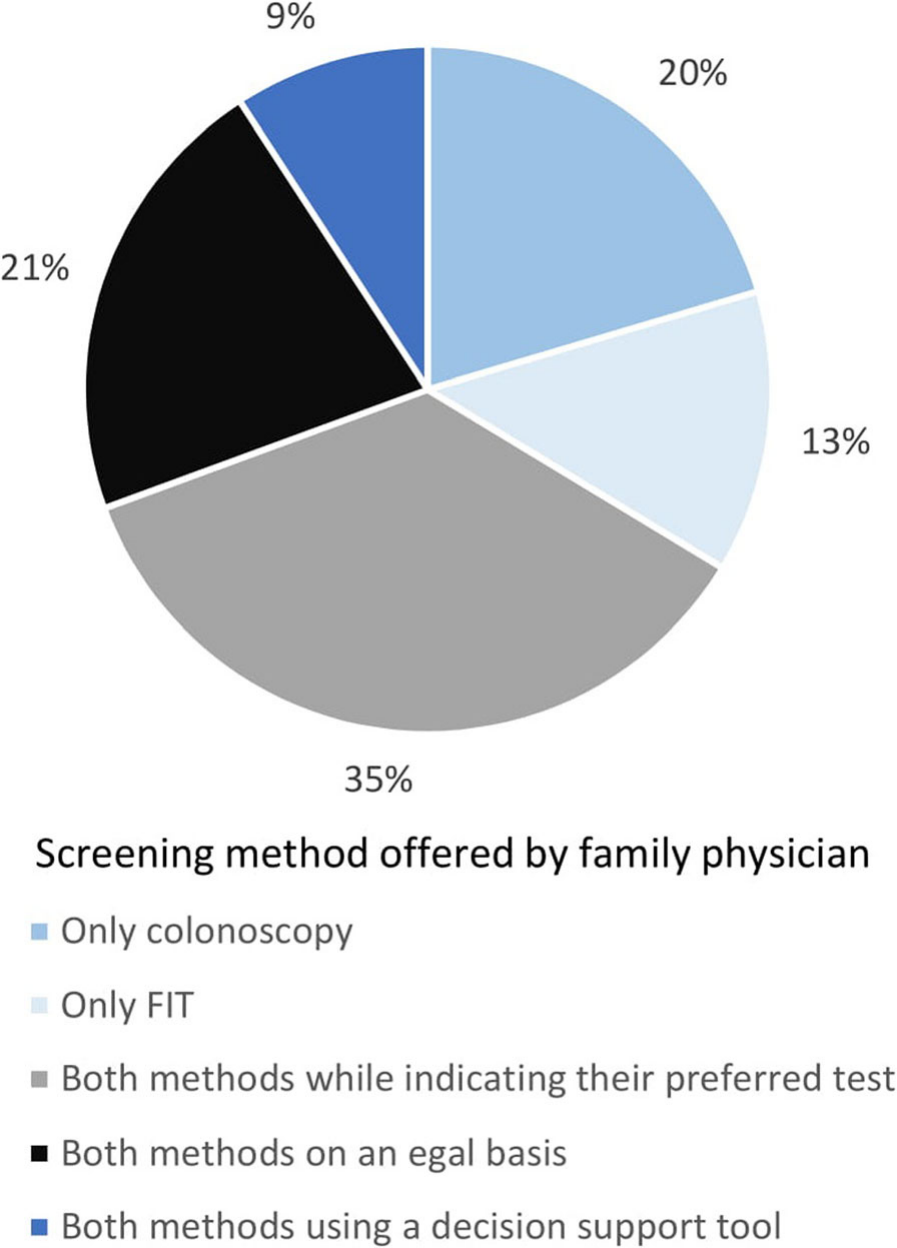
Colorectal cancer screening test offered by family physicians (*n* = 178)

Regarding the screening method offered by FP, 20% (*n* = 36) of FPs prescribed only colonoscopy, 13% (*n* = 23) only FIT, 35% (*n* = 62) both while indicating their preferred test, 21% (*n* = 37) both screening methods on an equal basis and 9% (*n* = 16) both methods using a decision support tool (Fig. 1). When asked how decisions about CRC screening were made in their practice, the majority of FPs surveyed (*n* = 108, 61%) reported that they took decisions with the patient on an equal basis.

When asked about the two main barriers that prevent FPs from informing and including patients in the program, they most often reported lack of time (*n* = 86, 48%). Second, they found the inclusion procedure too complex and time-consuming (*n* = 63, 36%). Third, they had patients already being followed by a gastroenterologist not participating in the program (*n* = 38, 21%), and finally fourth position, they just forgot to inform the patient (*n* = 15, 8%).

Conversely, when asked which factors most facilitated the discussion and/or inclusion of eligible patients in the program, the main facilitating factor was having more time (*n* = 121, 68%). Second, FP would like to see the tools for inclusion better adapted to their practice (*n* = 51, 29%). Third, FP would like to be better paid for this work (*n* = 19, 11%) and, finally, they need tasks to be better distributed among the various stakeholders in the program (*n* = 16, 9%).

The first model revealed no statistically significant correlations between the FPs reporting that they inform > 75% of their eligible patients and FP’s characteristics. Three FP’s characteristics had a significant association with offering colonoscopy only vs a choice of tests. With the multivariate logistic regression, the screening test the FP have done/would do for themselves (OR 8.54 [95% CI 1.83–39.79, *P* < 0.01]); second, being > 20 years in practice (OR 4.8 [95% CI 1.3–0.17.66, *P* = 0.02]); and third, seeing 300 or more patients per month (OR 3.05 [95% CI 1.23–7.57, *P* = 0.02]) were significant (Table 1).

Most of the FPs (75%; *n* = 131) were satisfied with the overall organization of the program and 71% (*n* = 125) were satisfied with the procedure for including a patient in the program regardless of the method used (on the program’s portal site or on the paper inclusion form). 87% (*n* = 155) of FPs believed they play an important or very important role in the implementation, specifically in the conduct of the program. As for the free-text question about improving program implementation, 19% (*n* = 31) of respondents indicated that patients should be better and more informed in advance through postal mail and large-scale communication campaigns.

The 10 FPs interviewed included 4 men and 6 women, half of whom were over 50 years old and the other half under 50 and included 1 who offered colonoscopy only and 9 who offered a choice of tests. During the semi-directive interviews, it emerged that 5 of 10 FPs interviewed informed the patient of the existence of the CRC screening program of canton of Vaud already at the first consultation: “In any case, I propose, I ask him if he has already done so and I inform him by saying: ‘You know, at the moment there is a program that offers screening’”. During regular follow-up with patients, all the physicians interviewed reported informing the patient of the existence of the program at least once. Regarding the screening method proposed, 6 out of 10 (60%) physicians interviewed proposed the two methods, however 2 (20%) of them influenced the patient to turn to colonoscopy. Finally, 3 (30%) FPs only offered colonoscopy “I’m more in favour of encouraging people to do a colonoscopy right away and not necessarily go through the FIT, because I think as long as they’re thinking about getting screened, you want to have the most effective screening possible right away”. One of the explanations given for this choice of method, colonoscopy, was the perceived low sensitivity of the FIT test. A FP aged over 50 said: “I am very for colonoscopy, I think that’s how we were “raised”. Before, we only had Guaiac for blood in the stool and they were really bad.” All but one of the FPs interviewed didn’t use decision aids during their consultations. The one using it said, «Yes I use it. It saves time ».

FPs were also asked about their role in the CRC screening program of canton of Vaud. All participants responded that their role was to inform patients about the program. The factor that made it easier to inform a patient for 6 (60%) FPs was if patients were informed of the program in advance of their consultation. Various obstacles were raised, such as the availability of gastroenterologists (*n* = 1, 10%), that sometimes they just forgot (*n* = 2, 20%) and a lack of time (*n* = 1, 10%).

## Discussion

This study found that the preventive practices of most FPs involved in the CRC screening program of the canton of Vaud were consistent with the program’s objectives namely, “informing patients of the program” and “presenting the choice of colonoscopy and FIT” and that the clear majority of FPs believed that their role was essential to the success of this program.

We noted that some FPs did not offer both screening methods to patients and that there was a preference for colonoscopy. As a result, screening rates might be lower than otherwise possible, because proposing a choice of screening methods or FIT only has been shown to improve screening rates in other settings [11, 12]. Having already had a colonoscopy was the strongest single predictor of offering only colonoscopy, even after adjusting for FP’s age and sex. It was found that 94% of FPs who prescribed colonoscopy had already performed this test themselves or would like to do it at over 50 years of age. This result could be expected since a study carried out in the US showed that FP’s preference for endoscopy was associated with patient endoscopic screening use [18].

Some age-related differences clearly reflected a generational evolution. Correlations showed that FPs with more than 20 years of experience were the ones who were more likely to prescribe colonoscopy instead of proposing both methods. The explanation given in the semi-directive interviews for this choice of method was the feeling of low sensitivity of the FIT test. A study in Toronto, Ontario, also found that a group of FPs in the sample strongly favoured colonoscopy, believing that faecal occult blood testing was a less effective method [19]. The explanation for this is that previously, until 2015 in Vaud, blood in stool was primarily tested using the Guaiac Faecal Occult Blood Test (G-FOBT), which had a sensitivity of only 50% and was not specific to human haemoglobin. With the organized program, blood testing in the stool is done through the FIT test, which has a sensitivity between 70 and 85% and is specific for human haemoglobin and colorectal bleeding. The analysis of semi-quantitative FIT is automated, and allows reliable and reproducible interpretation [20, 21]. A study in Brazil also showed that the longer FP have been in practice the less they prescribe colorectal cancer screening [22]. Furthermore, regarding the method of screening, almost all of them perceived colonoscopy as being very effective in reducing CRC mortality and were less certain, however, about the effectiveness of FIT [22]. Further efforts to promote shared decision making in the program should specifically target older FPs.

Finally, the number of patients seen by a FP per month also influenced the FP’s proposal of a screening method to the patient. Those who saw less than 300 patients per month were more likely to offer both screening methods. FPs who saw fewer patients may have had more time to spend with each patient. This allowed them to explain the two screening methods and give the patient the choice. It would be interesting in a future study to explain this difference by looking at billing for CRC screening counselling. Another study conducted in the US supported the same result since it showed that FPs with smaller patient volumes (< 100 patients per week) were more likely to have patients reporting enough time to discuss CRC with their FP [18]. But, a study conducted in Brazil revealed that there was no significant influence of the number of patients seen per week and attitudes by screening practice, specifically lack of screening practice [22].

Lack of time was the major factor that influenced the differences between FP practices and the objective of the program to “inform patients of the program”. Regarding this barrier, the majority of doctors noted that it would be appropriate for all eligible persons to be systematically informed of the existence of the program through a postal letter or a large-scale communication campaign. This recommendation, also raised during a survey on colorectal cancer screening in Geneva [23], would also inform the entire population and thus obtain egalitarian information. It is important to note that invitations to the program are being progressively implemented, with the goal of all citizens being invited every 2 years beginning at 50 years of age. A simplification of the inclusion procedure that would reduce the time of inclusion could also be an answer to the problem of lacking time.

The implication of these results for the CRC screening program of canton of Vaud is that FPs should be trained in both screening methods and shared decision-making consultations so that the FP’s personal opinion has less influence on his or her method proposals and so that FPs with more years of experience can become familiar with the FIT test.

The strong point of this study was the response rate to the online survey, which was 40%, a relatively high rate compared to other similar surveys conducted in the Canton of Geneva (19.4%), Neuchâtel and Jura (29%) and Valais (36%) [23]. In addition, the survey of this study served to remind FPs of the existence of the colorectal cancer screening program of canton of Vaud. Three out of 10 FPs interviewed felt the survey had a positive impact on their intention to inform their patients about the screening program.

However, some limitations of this study should be noted. First, since participation was voluntary, we may have a selection bias as FPs’ interest in prevention in terms of colorectal cancer screening might have been a driving force for participating in the survey. Further, we limited our sample to the 89% of FPs registered with the program; however, even if we assume that the additional 11% do not inform 75% of their patients, we would still have greater than 50% of FPs informing 75% of the patients, our pre-determined threshold. Some results are likely to be overestimated due to desirability bias, especially as we relied on FP reporting and not actual practice. However, regarding this desirability bias, the FPs interviewed were very direct and honest. Another limitation of this study was that the survey was conducted with variables in dichotomous categories, limiting precision. If we had conducted this survey with continuous variables such as age, we might have had stronger associations with the outcomes of interest. Finally, differences between physicians may be confounded by differences in the composition of their patient panel, as patients’ conditions, illnesses, patients’ demographic condition, and population characteristics may affect FPs’ choices.

## Conclusion

In conclusion, FPs report taking their role in this program seriously and providing care consistent with the program objectives. In order to ensure equal information and to gain time, patients should be systematically informed in advance by postal mail and large-scale communication campaign. It is planned that the information brochure will be sent to the entire eligible population. However, at present, coverage is not complete. The program starts gradually over 5 to 7 years and the mailings are done in a segmented way. In addition, in order to increase the participation rate, it would be important for FPs to offer both screening methods to patients. To encourage this, physicians should be trained in both screening methods and in the shared decision-making that allows the patient to make a free and informed choice. Finally, in order for FPs to have more time available for patients to explain the two screening methods, better reimbursement could reduce pressure to see high volumes of patients.

## Supplementary information

**Additional file 1.** Survey questions, survey questions on surveygizmo.com used for the quantitative results.

**Additional file 2.** Interview guide.

## Data Availability

The questionnaire and the datasets used and analysed during the current study are available from the corresponding author on request.

## Abbreviations

CI: Confidence intervals
CRC: Colorectal cancer
FIT: Faecal immunochemical test
FP: Family physician
FPs: Family physicians
FVDC: Fondation vaudoise pour le dépistage du cancer
OR: Odds ratios
US: United States
WHO: World Health Organization
