# Supplementary material for: Self-reported screening practices of family physicians participating in the colorectal cancer screening program of the canton of Vaud: a cross-sectional study
Source: BMC Fam Pract. 2020 Jun 10;21:103. doi: 10.1186/s12875-020-01176-z (PMC7285614; doi:10.1186/s12875-020-01176-z)
Supplement: Supplementary file 1 — Additional file 1. Survey questions, survey questions on surveygizmo.com used for the quantitative results. [file 12875_2020_1176_MOESM1_ESM.docx]

Additional file 1: Survey questions, survey questions on surveygizmo.com used for the quantitative results

#### **STUDY ON THE ROLE OF FAMILY DOCTORS INVOLVED IN THE VAUDOIS COLORECTAL CANCER SCREENING PROGRAMME**

#### **This study aims to evaluate the implementation of the cantonal colorectal cancer screening programme from the perspective of general practitioners, by analysing its strengths and weaknesses, as well as the elements favouring and hindering the implementation of this screening in primary care medicine. In order to facilitate your practice and to improve the programme's progress, we would like to gather your experience.**

#### **The only condition for participation is to have included at least one patient in the cantonal colorectal cancer screening programme since 2015.**

#### **This study is being carried out as part of a Master's thesis in Public Policy and Management at the University of Lausanne, IDHEAP. It is coordinated by student Camille Ducrey and supervised by Prof. Cornuz, Prof. Horber-Papazian and Dr. Gouveia in collaboration with Dr. Ducros.**

#### **Participation in this study is on a voluntary basis. You have the right not to participate or to stop your participation at any time, without any justification. Participation in this study does not require any specific skills. We would like a representative sample of family doctors who have included at least one patient in the program since 2015 to participate. The data will be treated anonymously and confidentially.**

#### **If you would like more information about the study, you can contact us by e-mail: camille.ducrey@unil.ch.**

#### 1) I have included at least one patient in the cantonal colorectal cancer screening programme since 2015. After reading the above information, I voluntarily agree to complete this questionnaire..

( ) I accept

( ) I refuse

**Page entry logic:** This page will show when: #1 Question " I have included at least one patient in the cantonal colorectal cancer screening programme since 2015. After reading the above information, I voluntarily agree to complete this questionnaire." is one of the following answers ("I accept")

Questions relating to the cantonal colorectal cancer screening programme

*2) How did you find out about the colorectal cancer screening program in the Canton of Vaud?*

( ) Information from the Fondation vaudoise pour le dépistage du cancer (FVDC) or the University Medical Polyclinic (PMU)

( ) Information from colleagues

( ) Information by patients

( ) Other, please specify: ______*

*3) In your opinion, does the program meet a societal health need?*

( ) Yes

( ) Partly, why?: _____*

( ) No, why?: ____*

( ) No notice

*4) Are you satisfied with the overall organization of the program?*

( ) Yes

( ) Partly, why?: ______*

( ) No, why?: ______*

( ) No notice

**Logic: Show/hide trigger exists.**

*5) Have you been briefed on the program?*

( ) Yes

( ) No

**Logic: Hidden unless: #5 Question " Have you been briefed on the program ?" is one of the following answers ("Yes")**

*6) Are you satisfied with the information provided at these sessions?*

( ) Yes

( ) Partly, why?: ______*

( ) No, why?: ______*

*7) Are you satisfied with the process of including a patient in the program?*

( ) Yes

( ) Partially, why?: _______*

( ) No, why?: _______*

( ) No opinion

*8) What role do you see yourself playing in the implementation, more specifically in the running of the programme?*

( ) Very important

( ) Important

( ) Not very important

( ) Useless

( ) Other, please specify: ______*

*9) How are decisions about colorectal cancer screening made in your practice?*

( ) I make my own decisions

( ) I make my own decisions, taking into account the patient's opinion.

( ) I make the decision with the patient on an equal footing...

( ) The patient makes the decision

*10) On average, at what rate do you inform your eligible patients* of the existence of the program within the first 3 consultations? (*patients residing in the canton of Vaud between 50 and 69 years old)*

( ) 100% of patients presenting for a consultation and eligible for the programme

( ) 75% of patients presenting for a consultation and being eligible for the programme

( ) 50% of patients presenting for a consultation and being eligible for the programme

( ) 25% of patients presenting for a consultation and being eligible for the programme

*11) How many patients have you included in the program in the last six months?*

( ) 0

( ) 1

( ) Between 1 and 5

( ) More than 5

*12) What are the 2 barriers that most prevent and retain you from informing and including patients in the program?*

( )Lack of awareness of the program

( )Lack of knowledge of how to include a patient in the program

( ) Complex/timing inclusion procedure

Unclear guidelines for role definition

( ) Doubt about the effectiveness of the proposed tests

Insufficient remuneration for this work

( ) Lack of time

( ) Patient already being followed by a gastroenterologist not listed in the programme

( ) Other, please specify: _________

*13) What are the 2 factors that most facilitate the discussion and/or inclusion of eligible patients in the programme?*

( )Having more time

( ) Better defined roles

( ) Better communication between different stakeholders

( ) Better pay

( ) Better knowledge of the program

( ) Better knowledge of the inclusion procedure

( ) Better adapted means for inclusion

( ) Better training

( ) Other, please specify: ______

*14) When the patient decides to enroll in the program and decides to take a test, do you offer it to him/her:*

( ) More like a colonoscopy

( ) More like the FIT test

( ) Both, indicating your preference or past experience with either method.

( ) Both on an equal footing

( ) Both using a decision support tool.

( ) Others, to be specified: ______*

*15) What do you think could be improved in the implementation of this program?*

___________________________________________

Personal Questions

*16) In which type* of municipality do you work?*

* Criteria according to the BAG: https://www.bfs.admin.ch/bfs/fr/home/actualites/quoi-de-neuf.assetdetail.2543324.html

( ) Urban (63% of the resident population, 75% of the jobs and 16% of the surface area)

( ) Intermediate (21% of the resident population, 15% of the jobs and 24% of the surface area)

( ) Rural (16% of the resident population, 10% of the jobs and 60% of the surface area)

*17) How many years have you been in practice?*

( ) Less than 10 years

( ) Between 10 and 20 years old

( ) More than 20 years

*18) What is your activity rate?*

( ) 100%

( ) Between 50% and 100%

( ) 50% or less

*19) How many different patients do you see per month?*

( ) Less than 300

( ) Between 300 and 600

( ) More than 600

*20) Are you a man or a woman?*

( ) Male

( ) Woman

**Logic: Show/hide trigger exists.**

*21) To which age group do you belong?*

( ) Under 50 years old

( ) 50 years of age or older

**Logic: Hidden unless: #21 Question "** **To which age group do you belong ? " is one of the following answers ("Under 50 years old ")**

*22) When you are 50 years of age or older, will you be tested?*

( ) Yes, the method I would use is (Fit, Colonoscopy or Other): _____*

( ) No, I wouldn't want to be screened for colorectal cancer

**Logic: Hidden unless: #21 Question "** **To which age group do you belong ? " is one of the following answers ("50 years of age or older ")**

*23) Have you ever been tested?*

( ) Yes, the method I have used is (Fit, Colonoscopy or Other): ______*

( ) No, I haven't done a test yet, but I would like to do one (Fit, Colonoscopy or Other): ______*

( ) No, I don't want to be screened for colorectal cancer.

*24) How often do you exercise for a minimum of 20 minutes such as brisk walking, running or tennis?*

( ) Less than once a month

( ) Between 1 and 3 times a month

( ) More than 3 times a month

( ) Does not wish to answer this question

*25) How often do you drink alcohol?*

( ) Never

( ) Occasional (less than one drink a day)

( ) Regular (at least two drinks a day)

( ) Does not wish to answer this question

*26) What is your smoking status?*

( ) Non-smoker

( ) Ex-smoker

( ) Occasional use

( ) Daily consumption

( ) Does not wish to answer this question

*27) Which group does your diet correspond to?*

( ) A varied and balanced diet, 5 fruits and vegetables per day, less than 30% of total energy intake from fats and less than 10% of total energy intake from free sugars.

( ) Varied cuisine with fruits and vegetables

( ) Salty food, high fat and sugar content

( ) Does not wish to answer this question

*28) Would you be willing to be contacted again for a 30-minute one-on-one interview about the colorectal cancer screening program? If so, please note your e-mail address below so that we can contact you.*______

*Thank you!*

*Thank you for your participation in this survey. Your response is very important to us.*
