# Supplementary material for: Self-reported screening practices of family physicians participating in the colorectal cancer screening program of the canton of Vaud: a cross-sectional study
Source: BMC Fam Pract. 2020 Jun 10;21:103. doi: 10.1186/s12875-020-01176-z (PMC7285614; doi:10.1186/s12875-020-01176-z)
Supplement: Supplementary file 2 — Additional file 2. Interview guide. [file 12875_2020_1176_MOESM2_ESM.docx]

**Additional file 2: Interview guide**

Research introduction and presentation

1. Acknowledgement : Thank you for agreeing to conduct this interview and for taking part in the survey.

2. Research objectives: These interviews are part of a research project on the preventive practices of family doctors in the canton of Vaud involved in the Vaud colorectal cancer screening programme. They are conducted following an initial quantitative data collection which was done through an online survey of which you were also part. So these interviews are there to clarify some of the results of the survey.

3. Request for recording: with your agreement, I would like to record the interview. They will of course be destroyed after analysis. If you would like a copy of the recording, this is possible. Similarly, if you would like a copy of the survey results.

4. Anonymity: We guarantee anonymity, of course; your name will not appear, nor will any information that could identify you, such as place names or other information.

Development of the interview

Prevention :

5. First of all, I would like to ask you to tell me what prevention and preventive activities mean to you in general?

To deepen:

6. In your opinion, what is the most important area of prevention to address during a consultation?

7. What is the most difficult area of prevention to address in a consultation?

8. In your opinion, who is responsible for prevention?

Practice :

9. I would like to know if it would be possible to do a vignette together? A 55-year-old man coming in for a back pain consultation:

- Back pain due to osteoarthritis

- Smoking patient (1 pack per day)

- No high risk of colorectal cancer

10. How is the consultation generally conducted?

Do you address the issue of colorectal cancer screening, if so how?

11. How does the consultation take place when the topic of colorectal cancer screening is discussed?

12. How do you approach the choice of screening method?

If decision aid not used: what do you think of this table provided by the PMU?

Colorectal Cancer Screening Program :

13. I would like to discuss with you the role of family physicians in the colorectal cancer screening program. What can you tell me about it?

To deepen:

14. What do you think makes it easier for physicians to be effective in the role you've just described to me?

15. What do you think prevents doctors from being effective in the role you have just described to me?

To deepen:

In the survey it was found that the thing that most prevents or inhibits a physician from discussing or including a patient in the program is lack of time. What can you tell me about this?

16. Can you briefly describe to me the process of including a patient in the program, how does it work?

To deepen:

In the survey it was found that the second most common reason that physicians are unable to inform and include patients in the program is that the inclusion process is too complex and time consuming. What can you tell me about that?

Other:

17. Did the survey elicit anything new from you, a more particular interest in the topic, if so in what way?

18. If you were asked to give a message to other family physicians about this topic, what would it be?

19. If you were asked to give a message to the program organizers about this theme, what would it be?

20. We are coming to the end of the interview. I don't have any more specific questions to ask you, but perhaps there are other aspects that you would have liked to address that are important to you
